# Emerging role of exosome-derived non-coding RNAs in tumor-associated angiogenesis of tumor microenvironment

**DOI:** 10.3389/fmolb.2023.1220193

**Published:** 2023-08-04

**Authors:** Sai-Li Duan, Wei-Jie Fu, Ying-Ke Jiang, Lu-Shan Peng, Diabate Ousmane, Zhe-Jia Zhang, Jun-Pu Wang

**Affiliations:** ^1^ Department of General Surgery, Xiangya Hospital Central South University, Changsha, China; ^2^ Xiangya School of Medicine, Central South University, Changsha, China; ^3^ Department of Pathology, Xiang-ya Hospital, Central South University, Changsha, China; ^4^ Key Laboratory of Hunan Province in Neurodegenerative Disorders, Xiangya Hospital, Central South University, Changsha, China; ^5^ National Clinical Research Center for Geriatric Disorders, Xiangya Hospital, Central South University, Changsha, China

**Keywords:** exosomes, endothelial cells, exosomes-derived non-coding RNAs, tumorassociated angiogenesis, tumor microenvironment, lncRNA, miRNA, cancer

## Abstract

The tumor microenvironment (TME) is an intricate ecosystem that is actively involved in various stages of cancer occurrence and development. Some characteristics of tumor biological behavior, such as proliferation, migration, invasion, inhibition of apoptosis, immune escape, angiogenesis, and metabolic reprogramming, are affected by TME. Studies have shown that non-coding RNAs, especially long-chain non-coding RNAs and microRNAs in cancer-derived exosomes, facilitate intercellular communication as a mechanism for regulating angiogenesis. They stimulate tumor growth, as well as angiogenesis, metastasis, and reprogramming of the TME. Exploring the relationship between exogenous non-coding RNAs and tumor-associated endothelial cells, as well as their role in angiogenesis, clinicians will gain new insights into treatment as a result.

## 1 Introduction

The tumor microenvironment (TME), an intricate ecosystem, actively participates in every stage of cancer development (Hanahan and Weinberg, 2011; Yang et al., 2020). As a dynamic ecosystem containing a variety of cell types and non-cellular components, TME plays a major role in tumor growth, metastasis, and drug resistance. Cancers exhibit some biological behaviors, such as proliferation, migration, invasion, immune escape, angiogenesis, and metabolic reprogramming, all of which are affected by TME. Biological functions, including autocrine and paracrine functions, are regulated by the complex communication network within TMEs. Exocrine-mediated communication is an important emerging pathway of paracrine signal transduction (Giraldo et al., 2019). Exosomes can carry molecules such as *DNA*, *RNA*, and proteins to adjacent cells, where they act as effective signaling molecules between cancer cells and surrounding cells constituting TME. Nontumor cells in TME, such as fibroblasts, endothelial cells (ECs), and immune cells, are affected by tumor-associated active substances and their original cell functions undergo tumor-like changes, constantly adapting to the new environment and promoting tumor growth. The TME is composed of different cell types with various functions, which regulates excessive cell-cell interactions. These interactions orchestrate reprogramming to the environment allowed by each cancer and may have a significant impact on cancer development, progression, and treatment resistance.

**FIGURE 1:**
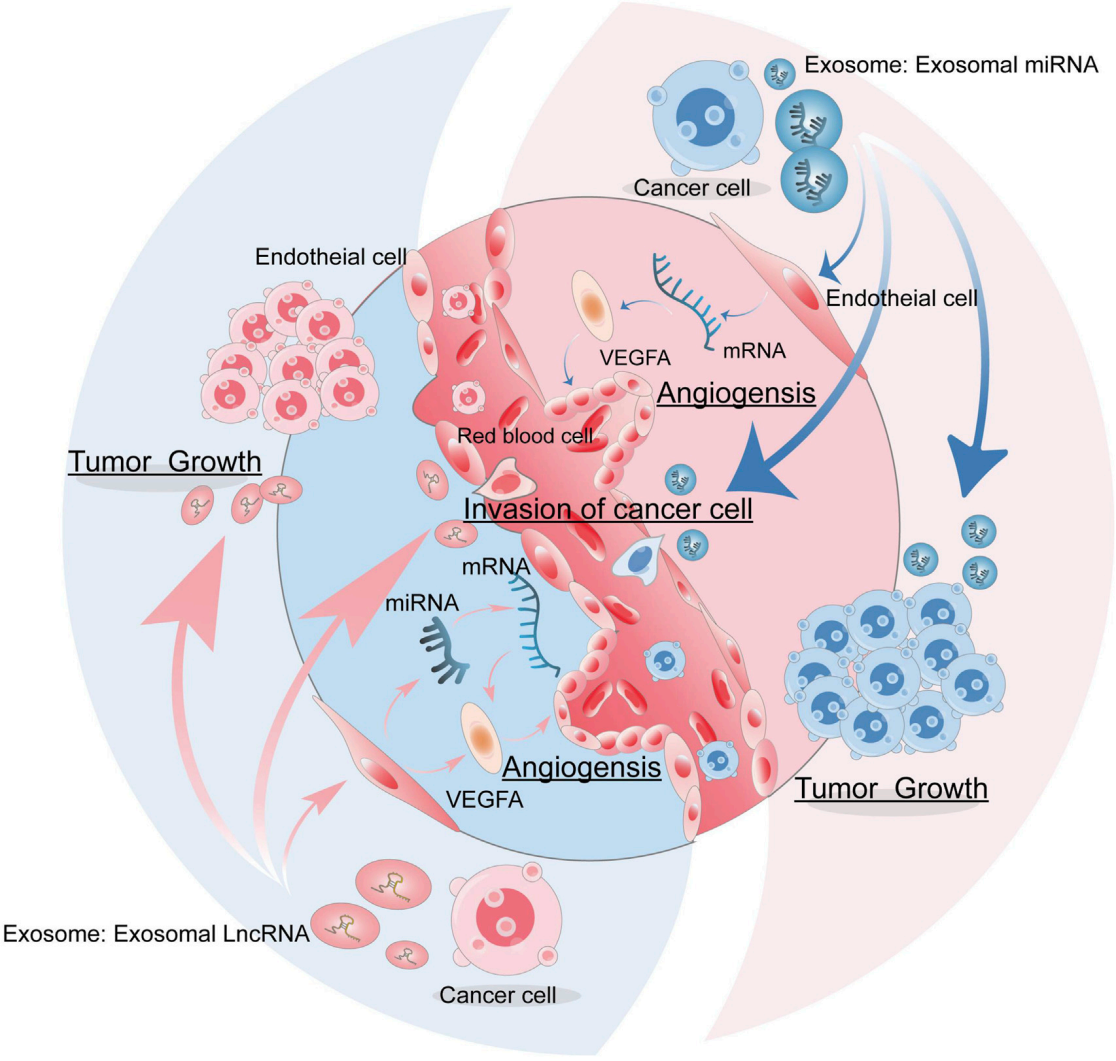
The role of exosomes in tumor angiogenesis. Tumor cells can release exosomes, which carry miRNA and lncRNAs that can act on endothelial cells to promote tumor angiogenesis, as well as stimulate tumor growth, invasion, and metastasis.

ECs are involved in tumor growth, tumor-induced angiogenesis, and vascular secretory functions for self-renewal and differentiation after trauma and thrombosis (Barachini et al., 2023). Angiogenesis plays an important role in all stages of cancer development (Aguilar-Cazares et al., 2019). Angiogenesis is a complex process of growing new capillaries from preexisting blood vessels, typically involving the following steps: stimulation of ECs with vascular endothelial growth factor (VEGF), proliferation, migration, and differentiation of vascular ECs, vessel branches and vessel formation (Ahir et al., 2020; Yang et al., 2022). Tumor vascular growth is a key factor in cancer progression, which is closely related to metastasis and a poor prognosis. Tumor angiogenesis is a recognized target for anticancer therapy by targeting growth factors, their cell surface receptors, and associated signaling pathways. Tissue hypoxia induces an overproduction of VEGF, leading to an imbalance between pro-angiogenic factors and anti-angiogenic factors, causing excessive abnormal angiogenesis that plays a central role in tumor progression (Jászai and Schmidt, 2019). The supply of energy and the removal of waste products are key factors in the development of cancer cells (Anderson and Simon, 2020). Tumor cells can communicate with adjacent tissues through the release of exosomes (Stec et al., 2015; Dominiak et al., 2020). Exosomes contain a variety of substances that promote angiogenesis and thus accelerate cancer invasion and metastasis (Głuszko et al., 2019), and the release of some exosomes also affects immune function (Aslan et al., 2019). Evidence suggests that non-coding *RNAs* (*ncRNAs*), especially long-chain non-coding *RNAs* (*lncRNAs*) and micro*RNAs* (*miRNAs*) in cancer-derived exosomes, play an important role in regulating angiogenesis by facilitating intercellular communication, which in turn stimulates tumor growth, as well as angiogenesis, metastasis, and reprogramming of TME (shown in Figure 1) (Zhao et al., 2020).

In hypoxic environments, hypoxia can induce overexpression of *ncRNA*, which is released by exosomes and participates in tumor angiogenesis by reacting with ECs and other angiogenic cells, thus affecting tumor progression (He et al., 2022; Jia et al., 2022; Yang et al., 2022). In addition to ECs, there are many other remaining cell-derived exosomes in TME that can also promote angiogenesis and thus help tumor cell metastasis. For example, exosomal *ncRNAs* released by tumor cells regulate ECs and promote or inhibit angiogenesis (Ahmadi and Rezaie, 2020). Tumor-associated macrophage (TAM)-derived exosomal *ncRNAs* regulate tumor cells and promote angiogenesis (Xu et al., 2022). Stem cells-derived exosomal *ncRNAs* regulate tumor cells and inhibit tumor angiogenesis (Yang and Teng, 2023). Tumor-derived exosomes (TEXs) of lung cancer cells can transfer *miR-21* to ECs *in vitro* and stimulate ECs angiogenesis to increase VEGF expression and secretion, thus helping to invade and metastasize lung cancer cells (Forder et al., 2021). The overexpression of exosome-derived *miR-16* and *miR-100* from mesenchymal stem cells downregulates VEGF expression in breast cancer cells, thus inhibiting angiogenesis and tumor growth *in vivo* and *in vitro* (Soheilifar et al., 2022). In hepatoma cells, cancer stem cells upregulate VEGF by delivering overexpressed *lncRNAH19* to ECs to promote angiogenesis and tumor growth (Yao et al., 2023). TAM-derived exosomes are enriched with *miR-501-3p*, which enhances the metastatic capacity of pancreatic ductal adenocarcinoma (PDAC) cells (Yin et al., 2019; Cocks et al., 2022). Blood vessel formation is inseparable from the role of ECs, and the relationship between exosomal *miRNAs* and *lncRNAs* and endothelial cells in TME is the focus of this review. By summarizing their relationship to explore the role of exogenous *ncRNAs* in tumor-associated endothelial cells and also their specific role in angiogenesis, clinicians will be able to gain new insights in cancer treatment.

## 2 Important position of exosomes

Previous studies have shown that a series of growth factors, cell surface receptors, and a large number of signaling molecules drive remodeling of the blood and lymphatic system in cancer (Stacker et al., 2014; Fares et al., 2020; Arcucci et al., 2021a). Recent studies have identified important roles for *ncRNAs* in the regulation of key aspects of cancer biology, including tumor angiogenesis and lymphangiogenesis. *NcRNAs* are a class of *RNA* molecules that do not encode proteins (Zampetaki et al., 2018), of which *miRNA* is the most studied, which along with *lncRNA* is the main focus of this review. *miRNAs* are small *RNA* molecules that mediate post-transcriptional regulation by targeting *mRNAs*, thereby resulting in the reduction of gene expression through *mRNA* degradation and/or translational repression. Nuclear *miRNAs* have been shown to play a role in transcriptional regulation through the recruitment of transcriptional activators and chromatin remodeling proteins of repressors (Bartel, 2009; Liu et al., 2018). It should be noted that different *miRNAs* can work together to focus on the expression of the same or multiple genes in related molecular pathways (Uhlmann et al., 2012). *LncRNA* exhibits a series of different regulatory functions in different cell compartments (Zampetaki et al., 2018). *LncRNA* plays a role in transcriptional regulation by binding chromatin remodeling proteins and recruiting transcription factors, activators, and inhibitors (Man et al., 2018).

Nuclear *miRNAs* can affect transcription by active ting or silencing of transcribed genes (Liu et al., 2018), and *miRNAs* participate in post-transcriptional processes by regulating *mRNA*. For example, *miR-29-b* regulates the expression of VEGFA and Akt3 by negatively inhibiting angiogenesis (Chen et al., 2017; Li et al., 2017). *LncRNA Hotair* can promote angiogenesis by directly activating the transcription of VEGFA genes (Fu et al., 2016). *LncRNA* can influence the cell cycle by regulating *mRNAs*. For example, *lncRNA MALAT1* can regulate the variable splicing of the carcinogenic transcription factor B-MYB in endothelial cells (Tripathi et al., 2013), *WTAPP1 lncRNA* promotes migration by increasing the expression of matrix metalloproteinase MMP1 (Li et al., 2018), and *tie-1As lncRNA* selectively binds and degrades *tie-1 mRNA*, leading to specific defects in cell connection and tube formation (Li et al., 2010). Furthermore, *lncRNA H19* regulates the biological behaviors of endothelial cells by suppressing *miR-29a*, thus inhibiting angiogenesis (Jia et al., 2016). *LncRNAs* facilitate epigenetic control of gene expression by recruiting transcription activators or inhibitors (Lam et al., 2013; Melo et al., 2013) or chromatin remodeling proteins as transcription regulators (Creamer and Lawrence, 2017). After gene transcription, *LncRNAs* can also be regulated, mainly by regulating mRNA splicing (Gong and Maquat, 2011), or by eliciting proteins that degrade *mRNAs* (Hutchinson et al., 2007) or acting as bait for proteins involved in *mRNA* degradation (Lee et al., 2016). *LncRNAs* can regulate various cancer-associated mRNAs by competitively sponging various *miRNAs*, and thus participate in relevant signaling pathways (Zhong et al., 2019). It is worth emphasizing that both *miRNAs* and *lncRNAs* can regulate the gene expression in complex biological responses: *miRNAs* regulate gene expression of proteins associated with their related molecular pathways by targeting *mRNAs*, and in addition, *miRNAs* can collaborate with other molecules to precisely mediate gene silencing. *LncRNAs* regulate gene expression by controlling chromatin remodeling, or by targeting *miRNAs* regulate gene expression by controlling chromatin remodeling or by targeting *miRNAs* (Guo et al., 2020; Mao et al., 2020).

### 2.1 The relationship between exosomal miRNAs and endothelial cells

Endothelial cells can form vascular systems to transport nutrients and metabolites, which can help tumor proliferation, invasion, and metastasis. Crosstalk stimulation between tumor cells and endothelial cells can promote the growth of both, improve tumor malignancy, and even develop resistance to treatment (Shweiki et al., 1992; Carmeliet and Jain, 2011). Tumor cells and certain immune cell subsets can promote angiogenesis by expressing and secreting growth factors or inducing hypoxia (Ding et al., 2014; Zhou et al., 2014), resulting in leakage of vascular structures that promote angiogenesis and metastatic spread of tumor cells. *MiRNAs* are endogenous *ncRNAs* consisting of 21–25 nucleotides that promote post-transcriptional regulation of target genes mainly by binding to the 3′untranslated region (UTR) of *mRNAs*. Meanwhile, *miRNAs* regulate more than 30% of gene expression in the body, and their functions are closely related to cell proliferation, differentiation, apoptosis, embryonic development, tissue and organ formation, as well as the occurrence and development of various diseases (Bartel, 2004). Recent studies have shown that exosome-mediated *miRNAs* transfer from cancer cells to endothelial cells, contributing to the breakdown of the endothelial cell barrier and allowing cancer cells to spread and metastasize to distant locations, such as cell-derived exosomal *miR-27b-3p* in colorectal cancer (Zhou et al., 2014; Dou et al., 2021). Furthermore, *miRNA*-containing exosomes from leukemia cells, such as *miR-17-92*, play an important role in communication between tumor and endothelial cells, thus regulating the process of tumor angiogenesis (Umezu et al., 2013).

**FIGURE 2:**
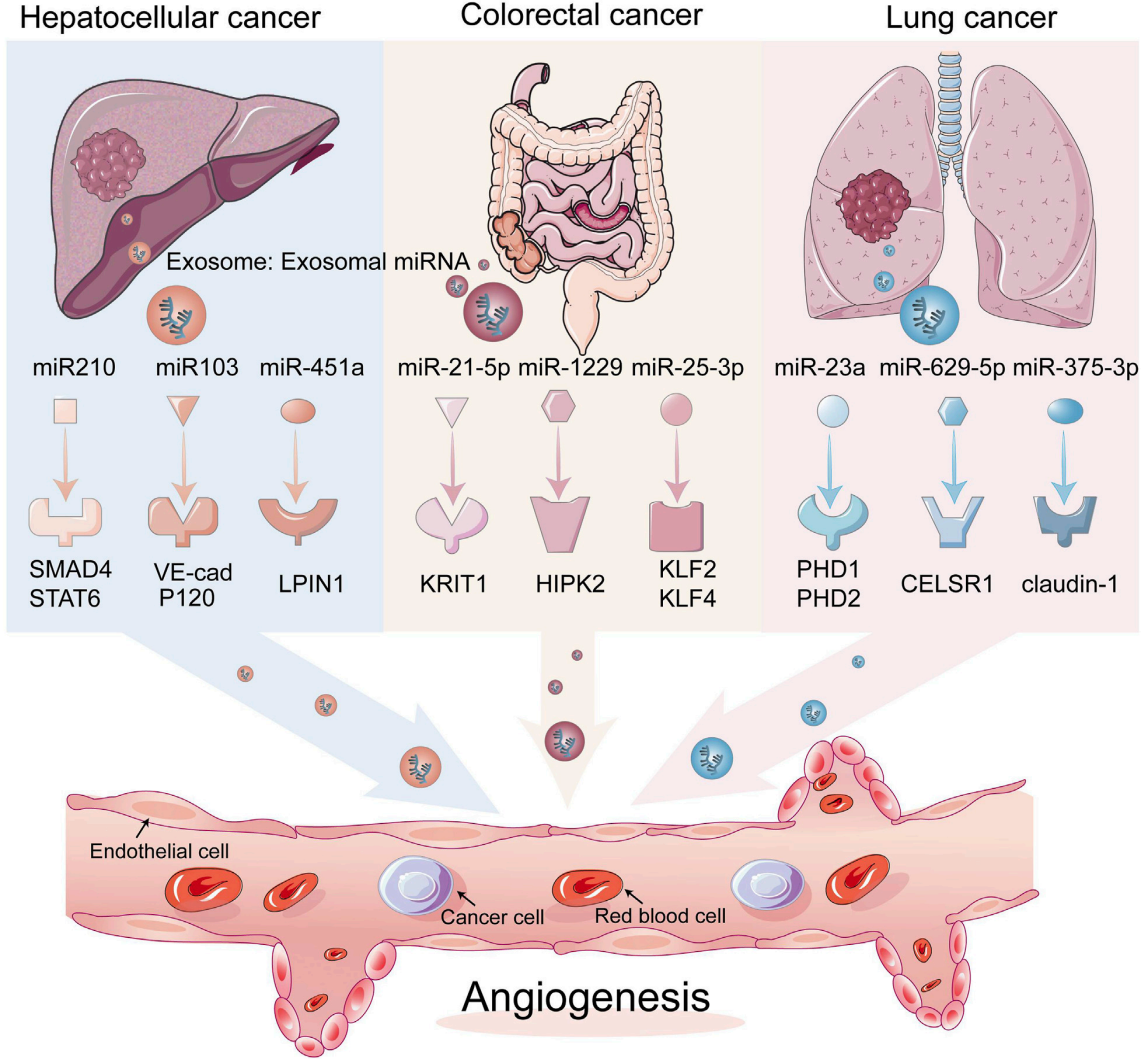
Exosomal miRNAs acting on endothelial cells affect tumor angiogenesis. Exosomal miRNAs can translocate from tumor cells to endothelial cells, which in turn acts on angiogenesis-related transcription factors, thereby stimulating angiogenesis.

Exosomal *miRNAs* can regulate the migration of tumor endothelial cells and the formation of lymphatic and blood vessels (Table 1; Figure 2). Within tumors, most exosomal *miRNAs* are thought to be produced by tumor cells (Huang et al., 2022). When internalized by endothelial cells, some of these *miRNAs* can stimulate angiogenesis or lymphangiogenesis by inhibiting the expression of proteins that inhibit the main pathways driving these processes (Duan et al., 2019; Kim et al., 2020; Masoumi-Dehghi et al., 2020). Exosomal *miRNAs* have been shown to downregulate several anti-angiogenic transcription factors in endothelial cells or inhibit the expression of VEGFA, a key inducer of angiogenesis, thus turning on the angiogenic switch (Li J. et al., 2020). For example, in gastric cancer, exosomal *miR-130a* and *miR-155* secreted by gastric cancer cells can inhibit the expression of the transcription factor c-MYB, indirectly promoting the expression of VEGFA (Arcucci et al., 2021b), which promotes angiogenesis and further assists in invasion and metastasis of gastric cancer cells.

**TABLE 1:** Relationship between exosomal miRNAs and angiogenesis in different types of cancer.

Types of cancers	MiRNAs in exosome	Roles of miRNAs in angiogenesis	Receptor cells	References
Hepatocellular carcinoma (HCC)	*miR-103*	Inhibiting the expression of VE cadherin	Endothelial cells	Fang et al. (2018)
	*miR-210*	Targeting Smad4 and STAT6	Endothelial cells	Lin et al. (2018a)
	*miR-1290*	Targeting SMEK1	Endothelial cells	Wang et al. (2021a)
	*miR-451a*	Targeting LPIN1	Endothelial cells	Zhao et al. (2019)
	*miR-638*	Down-regulating the expression of VE cadherin and ZO-1	Endothelial cells	Yokota et al. (2021)
	*miR-200b-3p*	Enhancing the expression of endothelial ERG	Endothelial cells	Moh-Moh-Aung et al. (2020)
	*miR-378b*	Directly promoting	Endothelial cells	Shi et al. (2021)
	*miR-296*	Being responsible for lymphangiogenesis	Endothelial cells	Shi et al. (2019)
Lung cancer (LC)	*miR-23a*	Inhibiting its targets PHD1 and 2	Endothelial cells	Hsu et al. (2017)
	*miR-629-5p*	Inhibiting CELSR1	Endothelial cells	Li et al. (2020b)
	*miR-30a-5p*	Inhibiting cell proliferation, migration and invasion abilities	Lung adenocarcinoma (LUAD) cells	Tao et al. (2021)
	*miR-141*	Targeting KLF12	Endothelial cells	Mao et al. (2020)
	*miR-375-3p*	Binding the 3′UTR of the tight junction protein claudin-1	Endothelial cells	Mao et al. (2021)
	*miR-486-5p*	Targeting the CADM1/tight junction axis	Endothelial cells	Sun et al. (2021)
Glioma (GBMLGG)	*miR-148a-3p*	Inhibiting ERRFI1 and activating EGFR/MAPK signaling pathway	Endothelial cells	Wang et al. (2020a)
	*miR-26a*	Targeting PTEN	Endothelial cells	Wang et al. (2019)
	*miR-21*	Via miR-21/VEGF signaling pathway	Endothelial cells	Mezzadra et al. (2017)
	*miR-944*	Inhibiting AKT/ERK signaling	Endothelial cells	Jiang et al. (2021)
	*miR-182-5p*	Inhibiting Kruppel-like factors 2 and 4	Endothelial cells	Li et al. (2020a)
Colorectal Cancer (CRC)	*miR-27b-3p*	Transferring to human umbilical vein endothelial cells	Endothelial cells	Dou et al. (2021)
	*miR-21-5p*	Transferring to human umbilical vein endothelial cells	Endothelial cells	He et al. (2021a)
	*miR-25-3p*	Targeting KLF2 and KLF4	Endothelial cells	Zeng et al. (2018)
	*miR-1229*	Targeting HIPK2	Endothelial cells	Hu et al. (2019)
Oral Squamous Cell Carcinoma (OSCC)	*miR-210-3p*	Targeting EFNA3 via PI3K/AKT pathway regulation	Endothelial cells	Wang et al. (2020b)
	*miR-130b-3p*	Inhibiting human umbilical vein endothelial cells	Endothelial cells	Yan et al. (2021)
	*miR-221-3p*	Targeting PIK3R1	Endothelial cells	He et al. (2021b)
cervical squamous cell carcinoma (CESC)	*miR-221-3p*	Targeting THBS2	Endothelial cells	Zhou et al. (2019)
	*miR-663b*	Inhibiting vinculin	Endothelial cells	You et al. (2021)
	*miR-142-5p*	Inducing IDO expression via ARID2-DNMT1-IFN-γ signaling	Lymphatic endothelial cells (LECs)	Zhou et al. (2021)
	*miR-221-3p*	Targeting VASH1	Endothelial cells	Wu et al. (2019)
	*miR-9*	Targeting MDK and modulating the PDK/AKT pathway	Endothelial cells	Lu et al. (2018)
	*miR-23a*	Inhibiting TSGA10	Endothelial cells	Bao et al. (2018)
Ovarian Cancer (OV)	*miR-205*	Via PTEN-AKT pathway	Endothelial cells	He et al. (2019)
	*miR-141-3p*	Activating JAK/STAT3 and NF-κB signaling pathways	Endothelial cells	Masoumi-Dehghi et al. (2020)
Pancreatic Cancer (PAAD)	*miR-27a*	Via BTG2	Endothelial cells	Shang et al. (2020)
Renal clear cell carcinoma (RCCC)	*miR-185-5p*	Binding to the promoter region of HIF2A mRNA	Endothelial cells	Braga et al. (2019)

#### 2.1.1 Hepatocellular carcinoma cells

Current studies have shown that in hepatocellular carcinoma (HCC), exosomal *miR-210* secreted by HCC cells can be transferred to endothelial cells, thus promoting tumor angiogenesis by targeting SMAD4 and STAT6 (Lin X. J. et al., 2018). *miR-1290* targeting SMEK1 promotes angiogenesis of hepatocellular carcinoma, and *miR-451a* targeting LPIN1 suppresses hepatocellular tumorigenesis by regulating tumor cell apoptosis and angiogenesis (Zhao et al., 2019; Wang Q. et al., 2021). HANR is responsible for lymphangiogenesis in HCC cells via the exosomal *miR-296* and the EAG1/VEGF axis (Shi et al., 2019). *miR-103* was delivered to ECs through exosomes and then attenuated the integrity of the endothelial junction by directly inhibiting the expression of VE-Cadherin (VE-Cad) (Fang et al., 2018). *miR-638* can promote vascular permeability by downregulating endothelial expression of VE-Cad and ZO-1 (Yokota et al., 2021). Exosomal *miR-200b-3p* from hepatocytes inhibited endothelial ERG expression, while reduction of *miR-200b-3p* in cancer cells promoted angiogenesis in HCC tissues by improving endothelial ERG expression (Moh-Moh-Aung et al., 2020).

#### 2.1.2 Colorectal cancer cells

The exosome *miR-21-5p* can be delivered from colon cancer cells to endothelial cells, targeting KRIT1 and thus inducing angiogenesis and vascular permeability, as can the exosome *miR-25-3p*, which also transfers to ECs and promotes CRC metastasis by targeting KLF2 and KLF4 to regulate growth factors in endothelial cells. Furthermore, there is *miR-1229* that promotes angiogenesis by targeting HIPK2 (Zeng et al., 2018; Hu et al., 2019; He Q. et al., 2021). *miR-27b-3p* is transferred by EMT-CRC cells into the exosomes of human umbilical vein endothelial cells (HUVEC), weakening the vascular barrier (Dou et al., 2021).

#### 2.1.3 Lung cancer cells

*miR-23a* directly inhibits its targets, prolyl hydroxylases 1 and 2 (PHD1 and 2), in exosomes from lung cancer cells, resulting in the accumulation of hypoxia-inducible factor 1 alpha (HIF-1α) in endothelial cells. Finally, hypoxic lung cancer cells enhanced angiogenesis through hypoxic cancer-derived exosomes under normoxic and hypoxic conditions (Hsu et al., 2017). For lung adenocarcinoma (LUAD), *miRNAs* affect cancer cells and ECs bidirectionally; for example, *miR-629-5p* in lung adenocarcinoma transfers to endothelial cells, and by inhibiting CELSR1, which is lower in endothelial cells in invasive LUAD (a *miR-30a-5p*, a non-canonical cadherin, increases endothelial monolayer permeability, while overexpression of *miR-30a-5p* in endothelial cells inhibited tumor development (Li et al., 2020b; Tao et al., 2021). The exosome *miR-141* is transported into HUVEC cells and targets KLF12 to promote angiogenesis in small cell lung cancer (SCLC), and *miR-375-3p* destroys vascular endothelial cells by directly binding to the 3′UTR of the tight junction protein CLDN1 and negatively regulating its expression tight junctions (Mao et al., 2020; Mao et al., 2021). *miR-486-5p* in non-small cell lung cancer (NSCLC) targets the CADM1/tight junction axis in vascular endothelial cells to promote metastasis of non-small cell lung cancer cells (Sun et al., 2021).

### 2.2 The relationship between exosomal lncRNAs and endothelial cells

**FIGURE 3:**
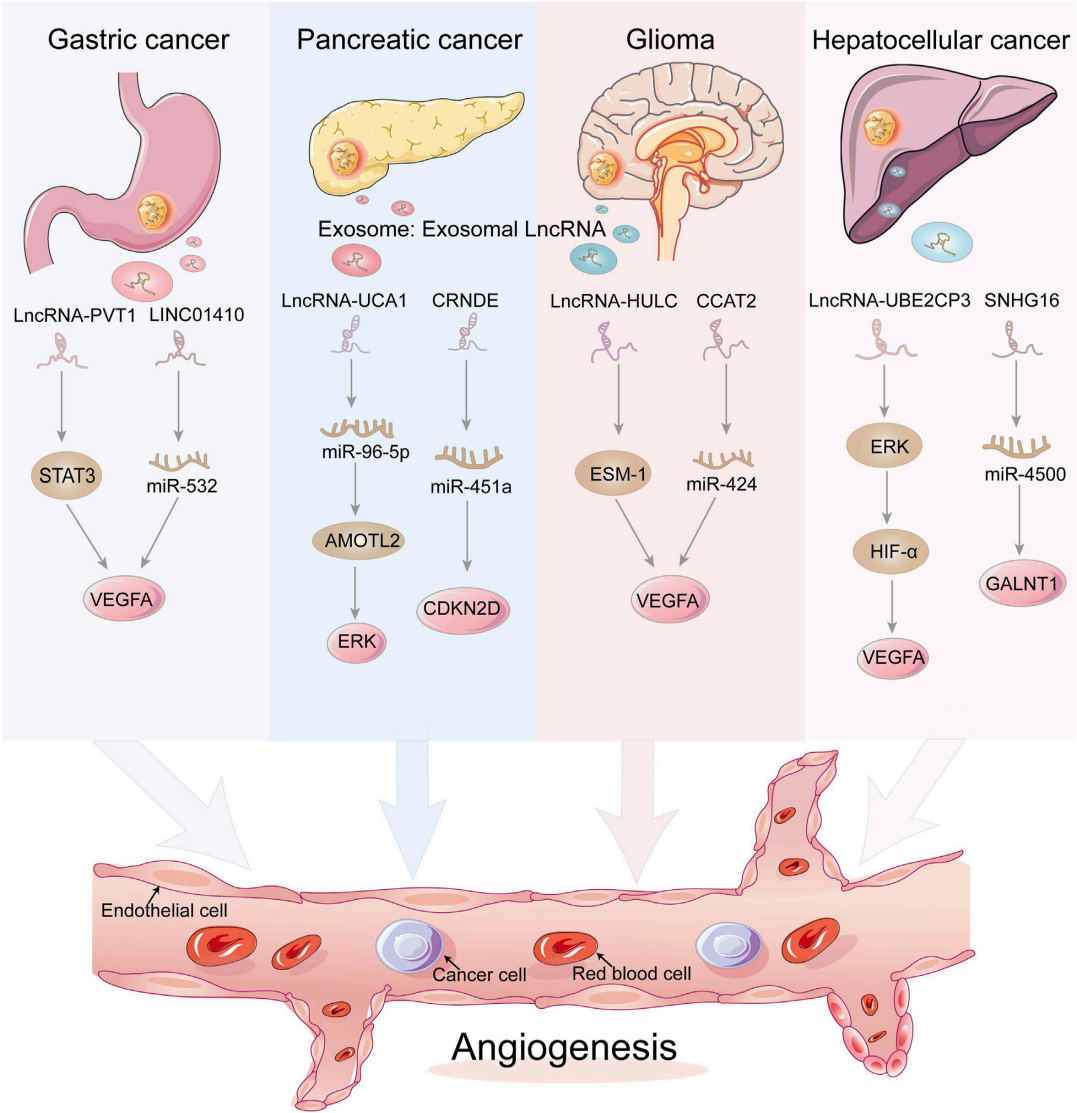
Exosomal LncRNAs act on endothelial cells to regulate tumor angiogenesis through a variety of mechanisms. Dysregulated lncRNAs can act directly as tumor factors or act on miRNAs at the transcriptional level, which in turn regulate tumor angiogenesis.

*LncRNAs* are a diverse class of transcribed RNA molecules that are more than 200 nucleotides llong and have limited protein coding potential (Nagano and Fraser, 2011; Spizzo et al., 2012). Current estimates from the GENCODE database (www.gencodegenes.org) suggest that the human genome contains approximately 16,000 *lncRNA* genes encoding over 28,000 distinct *lncRNAs*. Many *lncRNAs* have emerged as key players in the regulation of numerous biological processes in cancer, such as differentiation, cell cycle regulation, and immune responses (Guttman et al., 2009; Qiu et al., 2015; Bach and Lee, 2018). They can act directly as tumor suppressors or oncogenes, or be regulated by well-known tumor suppressors or oncogenes at the transcriptional or post-transcriptional level (Barsyte-Lovejoy et al., 2006; Huarte et al., 2010). ECs that line the inner surface of the blood vessels are an important part of the matrix in the TME (Junttila and de Sauvage, 2013; Kohlhapp et al., 2015). They are believed to be critical for angiogenesis and tumor metastasis, and *lncRNAs* may affect tumor progression by regulating endothelial cell biological behavior (Table 2; Figure 3). For example, *lncRNA H19* has been reported to be significantly upregulated in glioma-associated endothelial cells cultured in glioma-conditioned medium. Knockdown of *lncRNA H19* inhibited glioma-induced endothelial cell proliferation, migration, and tube formation *in vitro*. Mechanistic evidence suggests that *lncRNA H19* regulates the biological behavior of glioma-associated endothelial cells by inhibiting *miR-29a* (Jia et al., 2016). Furthermore, *lncRNA-APC1* plays an important tumor suppressor role in the pathogenesis of colorectal cancer. The following mechanistic studies show that *lncRNA-APC1* reduces exosome production in colorectal cancer cells by reducing *Rab5b mRNA* stability, and this effect inhibits tumor angiogenesis by inhibiting the over-activation of the MAPK pathway in endothelial cells (Wang F. W. et al., 2021). Dysregulated *lncRNAs* affect endothelial cell biological behavior through multiple mechanisms, so regulation of specific *lncRNA* expression in tumor cells or/and endothelial cells may have a significant impact on cancer progression.

**TABLE 2:** Ways of exosomal lncRNAs to promote angiogenesis in different types of cancer.

Types of cancers	LncRNAs in exosome	Ways of lncRNAs to promote angiogenesis	Receptor cells	References
Hepatocellular carcinoma (HCC)	*lncRNA UBE2CP3*	Activating the ERK/HIF-1α/p70S6K signaling cascade	Endothelial cells	Lin et al. (2018b)
	*lncRNA H19*	Affecting its tumor microenvironment	Endothelial cells	Conigliaro et al. (2015)
	*MALAT1*	Activating ERK1/2 signaling	Endothelial cells	Malakoti et al. (2021)
	*SNHG16*	Sponging miR-4500	Endothelial cells	Li et al. (2021)
	*lncRNA HULC*	Via VEGF and ESM-1	Endothelial cells	Zhu et al. (2016)
	*LncRNA-OR3A4*	Via AGGF1/akt/mTOR	Endothelial cells	Li et al. (2019)
Hepatoblastomas (HBs)	*lncRNA CRNDE*	Modulating mTOR signaling	Endothelial cells	Dong et al. (2017)
Gastric cancer (GC)	*lncRNA PVT1*	Inducing the STAT3/VEGFA axis	Endothelial cells	Zhao et al. (2018)
	*X26nt*	Binding to the 3′UTR of VE-cadherin mRNA	Endothelial cells	Chen et al. (2021)
	*LINC01410*	Depleting miR-532-5p	Endothelial cells	Zhang et al. (2018)
Non-Small Cell Lung Cancer (NSCLC)	*TNK2-AS1*	Enhancing STAT3 signaling through increasing VEGFA expression	Endothelial cells	Wang et al. (2018a)
	*lncRNA-p21*	Promoting tube formation and enhancing adhesion of tumor cells	Endothelial cells	Castellano et al. (2020)
	*lncRNA LINC01356*	Remodeling the blood-brain barrier	Endothelial cells	Geng et al. (2022)
	*Lnc-MMP2-2*	Targeting the miRNA-1207-5p/EPB41L5 axis	Endothelial cells	Wu et al. (2021)
Pancreatic Cancer (PAAD)	*lncRNA UCA1*	Through the miR-96-5p/AMOTL2/ERK1/2 axis	Endothelial cells	Guo et al. (2020)
	*CCAT1*	Binding to miR-138-5p to increase HMGA1 expression	Endothelial cells	Han et al. (2021)
Glioma (GBMLGG)	*lnc-POU3F3*	Secreting linc-POU3F3-enriched exosomes	Endothelial cells	Lang et al. (2017a)
	*lnc-CCAT2*	Activating VEGFA and TGFβ	Endothelial cells	Lang et al. (2017b)
Osteosarcoma (OS)	*EWSAT1*	Increasing secretion of angiogenic factors	Endothelial cells	Qiu et al. (2018)
	*lncRNA RAMP2-AS1*	Acting as a ceRNA of miR-2355-5p and regulating the expression of VEGFR2	Endothelial cells	Cheng et al. (2020)
	*MALAT1*	Blocking the pro-angiogenic effects potentially	Endothelial cells	Zhang et al. (2017b)
Breast cancer (BC)	*lncRNA AC073352.1*	Binding to YBX1	Endothelial cells	Kong et al. (2021)
	*MEG3*	Inactivating AKT signaling	Endothelial cells	Zhang et al. (2017c)
	*lncRNA GS1-600G8.5*	Reducing TEER and increasing BBB permeability	Endothelial cells	Lu et al. (2020)
Colorectal Cancer (CRC)	*lncRNA-APC1*	Activating the MAPK pathway	Endothelial cells	Wang et al. (2021b)
	*lncRNA PCAT1*	Regulating the activity of the miR-329-3p/Netrin-1-CD146 complex	Endothelial cells	Fang et al. (2022)
salivary adenoid cystic carcinoma (SACC)	*MRPL23-AS1*	Forming an RNA-protein complex with EZH2	Endothelial cells	Chen et al. (2020)
Nasopharyngeal carcinoma (NPC)	*CCAT2*	Via nasopharyngeal carcinoma-derived exosomal lncRNA CCAT2	Endothelial cells	Zhou et al. (2020)
Lung Adenocarcinoma (LAD)	*lncRNA LOC100132354*	Activating the VEGFA/VEGFR2/RAF/MEK/ERK signaling pathway	Endothelial cells	Wang et al. (2018b)
Epithelial ovarian cancer (EOC)	*MALAT1*	Transferring to recipient HUVECs and affecting HUVECs	Endothelial cells	Qiu et al. (2018)
Bladder cancer (BCa)	*lncRNA BCYRN1*	Enhancing VEGF-C/VEGFR3 signaling-induced BCa lymphatic metastasis	Endothelial cells	Lei and Mou (2020)
Cervical Cancer (CC)	*TUG1*	Being transferred to the recipient HUVEC	Endothelial cells	Tao et al. (2020)
Thyroid Cancer (TC)	*FGD5-AS1*	Targeting the miR-6838-5p/VAV2 axis	Endothelial cells	Liu et al. (2022)

#### 2.2.1 Gastric cancer cells

*PVT1* is an oncogenic *lncRNA* that is significantly expressed in gastric cancer, especially in patients with low differentiation and progressive stages. *PVT1* can bind to different proteins to exert oncogenic effects, and in gastric cancer, *PVT1* can bind to the signal transduction activator STAT3 to ensure that it is not degraded, thus activating the STAT3 signaling pathway and thus increasing VEGFA in gastric cancer, thus activating the STAT3 signaling pathway and increasing the expression of VEGFA to promote gastric cancer angiogenesis. At the same time, activated STAT3 can also occupy the promoter of *PVT1* and promote *PVT1* expression, forming a positive feedback regulation (Zhao et al., 2018). Similarly, in NSCLC, the *lncRNA TNK2-AS1* can also bind to STAT3 to inhibit its degradation, thus activating the STAT3 signaling pathway and promoting tumor progression and angiogenesis. In addition, STAT3 can also bind to the *lncRNA TNK2-AS1* promoter to promote its transcription in positive feedback (Wang et al., 2018a). *LINC01410* is also one of the molecules that promote angiogenesis in gastric cancer. *LINC01410* can inhibit *miR-532-5p* expression, while silencing *miR-532-5p* reduces inhibition of NCF2, thus upregulating NCF2 expression and activating the NF-κB signaling pathway, exacerbating malignant progression and angiogenesis of gastric cancer. Interestingly, NCF2 can bind to the *LINC01410* promoter, thereby promoting its transcription, forming a positive feedback loop that exacerbates the development of gastric carcinogenesis (Zhang et al., 2018).

#### 2.2.2 Pancreatic cancer cells

An important feature of the tumor microenvironment is hypoxia caused by inadequate oxygen flow and abnormal tumor vasculature, and exposure of cancer cells to conditions of oxygen deficiency increases the release of exosomes, which in turn promotes angiogenesis and tumor metastasis. In hypoxic PC cells, the expression of *lncRNA UCA1* increases and can be transferred to human microvascular endothelial cells HUVECs, promoting angiogenesis and tumor growth via the miR-96-5p/AMOTL2/ERK1/2 axis (Guo et al., 2020). In addition to this, PC cell-derived exosomal *CRNDE* enhanced angiogenesis by binding to *miR-451a* to increase *CDKN2D* expression (Zhu et al., 2021).

#### 2.2.3 Glioma cells

One of the keys to glioma development is abnormal generation of tumor blood vessels, and high-grade gliomas clearly have a higher density of tumor blood vessels that contribute more to tumor development than low-grade gliomas. It has been shown that glioma cells can regulate the tumor microenvironment by secreting exosomes, for example, glioma exosomes can promote angiogenesis by transferring *LINC-POU3F3* to human brain microvascular endothelial cells (HBMEC) (Lang et al., 2017a). Additionally, *LINC-CCAT2* was found to be highly expressed in glioma cells U87-MG and could be transferred to HUVECs to activate the production of the angiogenic factors VEGFA and TGFβ, while inhibiting the expression of the apoptotic molecules Bax and caspase-3, thus promoting angiogenesis and inhibiting apoptosis in glioma cells (Lang et al., 2017b). *LncRNA HULC* is one of the most common oncogenes with the potential to promote invasion and angiogenesis. In glioma, Zhu Yu et al. showed that HULC can activate the PI3K/AKT/mTOR signaling pathway, which in turn regulates downstream angiogenic factors VEGF and ESM-1. Furthermore, in a hypoxic environment, HULC can upregulate HIF-1α, which is also one of the key molecules that promote the secretion of angiogenic factors (Zhu et al., 2016).

#### 2.2.4 Hepatocellular carcinoma cells

As tumor growth requires more and more nutrients, this requires the secretion of angiogenic substances to promote tumor angiogenesis. *LncRNA* has been shown to regulate ECs function and promote the expression of angiogenic factors to regulate angiogenesis. Lin et al. demonstrated that the *lncRNA UBE2CP3* can activate the ERK/HIF-1α/p70S6K signaling pathway, increase VEGFA expression and regulate ECs function, thus promoting angiogenesis in hepatocellular carcinoma (Lin J. et al., 2018). Cancer stem-like cells, also known as CD90^+^ hepatocellular carcinoma cells, are enriched in *lncRNA H19*, which can be released by encapsulating in exosomes and then transported to endothelial cells, promoting the expression of the angiogenic factor VEGF in endothelial cells and thus regulating hepatocellular carcinoma angiogenesis (Conigliaro et al., 2015). Direct exosomal transfer of *MALAT1* to hepatocytes leads to increased invasion and migration of hepatocytes through activation of extracellular signal-regulated kinase 1/2 (ERK1/2) signaling (Li et al., 2020c). Exosomal *SNHG16* increases *GALNT1* expression by sponging *miR-4500* to promote angiogenesis. The *SNHG16/miR-4500/GALNT1* axis plays an important role in exosome-mediated angiogenesis and tumor growth *in vitro* and *in vivo* (Li et al., 2021). Furthermore, elevated expression of *lncRNA-OR3A4* in hepatocellular carcinoma is associated with angiogenesis and promotes the tube formation capacity of HUVEC, mainly through activation of the AGGF1/AKT/mTOR pathway (Li et al., 2019). *CRNDE* is upregulated in many tumors, promotes cell growth and migration, and is a recognized oncogene, also in hepatoblastoma. *CRNDE* knockdown inhibits tumor angiogenesis and reduces cell viability in hepatoblastoma, primarily through regulation of mTOR signaling (Dong et al., 2017).

#### 2.2.5 Other cancer cells

Some other cancer exosomal *lncRNAs* are still associated with endothelial cells (Table 2). Osteosarcoma originates from bone and is the most common of primary malignancies. Zhang et al. showed that *lncRNA MALAT1* is associated with osteosarcoma angiogenesis and hypoxic response and that *MALAT1* activates the mTOR/HIF-1α pathway, thereby promoting the production of angiogenic factors (Zhang Z. C. et al., 2017). In lung adenocarcinoma, the *lncRNA LOC100132354* can affect the downstream target gene VEGFA to promote tumor angiogenesis (Wang et al., 2018b). Some non-angiogenic *lncRNAs* have the ability to inhibit angiogenesis. For example, *GAS5* can inhibit the activation of the Wnt/β-catenin pathway to suppress angiogenesis in CRC (Song et al., 2019). Regarding MEG3, a recognized tumor suppressor, it inhibits tumor progression in breast cancer mainly by suppressing AKT signaling and also inhibits capillary angiogenesis in endothelial cells by reducing the expression of tumor angiogenic factors (Lu et al., 2020). The *lncRNA MALAT1* can be transported through exosomes to endothelial cells in epithelial ovarian cancer (EOC) and then regulates the vasculature of endothelial cells by generating related genes that stimulate pro-angiogenic behavior. In addition, serum exosomal *MALAT1* levels were strongly associated with advanced and metastatic outcomes, which were independent predictors of overall survival in EOC (Qiu et al., 2018). Interestingly, *lncRNAs* can affect exosome production in addition to being transported by exosomes. In colorectal cancer, activation of the Adenomatous Polyp in Colon (APC) gene of *lncRNA* (*lncRNA APC1*) can directly affect the stability of *Rab5b mRNA*, thereby inhibiting exosome production by CRC cells and ultimately tumor angiogenesis (Wang F. W. et al., 2021). Moreover, exosomal *lncRNA PDAT1* regulates the activity of the *miR-329-3p*/*Netrin-1-CD146* complex to promote tumor metastasis (Fang et al., 2022). In lung cancer, the exosomal *lncRNA LINC01356* and the exosomal *lnc-MMP2-2* derived from NSCLC cells play a key role in the remodeling of the blood-brain barrier, thereby participating in brain metastasis (Geng et al., 2022). Exosomal *lnc-MMP2-2* promotes brain metastasis via the *miRNA-1207-5p*/*EPB41L5* axis (Wu et al., 2021). In thyroid cancer, exosome *FGD5-AS1* targets the *miR-6838-5p*/*VAV2* axis to promote angiogenesis and metastasis (Liu et al., 2022).

## 3 Conclusion and prospect on endothelial cells and exosomes

Exosomes are important carriers of cell-to-cell communication signals and genetic material in the tumor microenvironment. In this review, we divide them into different types of cancer and summarize the relationship between *miRNAs* and *lncRNAs* with endothelial cells, promoting tumor angiogenesis and tumor angiogenesis. Mechanisms of lymphangiogenesis, demonstrating the complexity of their mediated angiogenesis in cancer development. Although *ncRNAs* do not encode proteins, they do play critical roles in regulating the levels of many cellular and extracellular proteins, particularly in the early stages of certain tumors, by mediating gene silencing at the transcriptional level to regulate the expression of cancer-related proteins, which in turn affects aspects of angiogenesis, apoptosis, and tumor metastasis. *NcRNAs* can be used as a new class of markers for early clinical diagnosis and prognosis, and exosomes can be used as carriers to deliver them to various parts of the body, helping them participate more actively in intercellular communication and function. Cancer-derived exosomal *ncRNAs* can promote tumor angiogenesis and lymphangiogenesis by altering gene expression in a vatiety of cell types, including endothelial cells. Therefore, the regulatory functions of *ncRNAs* in tumor angiogenesis and lymphangiogenesis can be considered multidimensional.

The mechanistic summary in this paper can help develop effective and precise cancer therapies and, based on current research related to the regulation of tumor angiogenesis by *ncRNAs*, can be used to develop new cancer biomarkers and therapies depending on the type of cancer. Identifying the different mechanisms involved in identifying therapeutic approaches has seminal implications for new cancer treatments, and more research is needed to achieve this. In addition, certain specific *ncRNAs* can be used as a new class of markers for early clinical diagnosis and prognosis, also providing a new idea for tumor treatment. *LncRNAs* and *miRNAs* may be a feasible strategy to monitor the efficacy of anti-angiogenic therapy and predict prognosis. In addition, regulation of angiogenesis-related signaling pathways may also serve as a new therapeutic direction, and the molecular mechanisms of *miRNAs* and *lncRNAs* in tumor development and development need to be investigated in more depth, thus contributing to the improvement of tumor diagnosis and treatment.

## Abbreviations

TME, Tumor microenvironment; ECs, endothelial cells; VEGF, vascular endothelial growth factor; ncRNAs, non-coding RNAs; lncRNAs, long-chain non-coding RNAs; miRNAs, microRNAs; TAM, Tumor-associated macrophage; TEXs, Tumor-derived exosomes; PDAC, pancreatic ductal adenocarcinoma; UTR, untranslated region; VASH2, Vasohibin 2; HCC, hepatocellular carcinoma; VE-Cad, VE-Cadherin; CRC, colorectal cancer; HUVEC, human umbilical vein endothelial cells; PHD1 and 2, prolyl hydroxylases 1 and 2; HIF-1α, hypoxia-inducible factor 1 alpha; LUAD, lung adenocarcinoma; SCLC, small cell lung cancer; NSCLC, non-small cell lung cancer; HBMEC, human brain microvascular endothelial cells; ERK1/2, extracellular signal-regulated kinase 1/2; EOC, epithelial ovarian cancer; APC, Adenomatous Polyp in Colon; EOC, epithelial ovarian cancer.
